# Non-traumatic Iliacus Muscle Hematoma During Anticoagulation Bridging in a Patient With Active Malignancy: A Case of Multifactorial Bleeding Risk

**DOI:** 10.7759/cureus.109221

**Published:** 2026-05-19

**Authors:** Samuel Fernandez Perez, Fernando A Sosa, Reubyn William Chong

**Affiliations:** 1 Internal Medicine, McGovern Medical School at UTHealth Houston, Houston, USA

**Keywords:** anticoagulation, bleeding risk, iliacus hematoma, malignancy, occult bleeding, warfarin, warfarin

## Abstract

Patients with malignancy are at increased risk of bleeding due to tumor-related vascular fragility, chemotherapy-induced thrombocytopenia, impaired hepatic function, malnutrition, and systemic inflammation. Anticoagulation management in this population is further complicated by frequent medication changes, drug-drug interactions, poor oral intake, and acute illness, making bridging anticoagulation a particularly high-risk transition. We report a case of a non-traumatic iliacus muscle hematoma occurring during therapeutic-dose enoxaparin bridging in a patient with active metastatic malignancy, illustrating how multiple concurrent risk factors can precipitate clinically significant hemorrhage even when the international normalized ratio (INR) appears subtherapeutic or within range.

A 67-year-old man with metastatic squamous cell carcinoma of the lung and larynx, atrial flutter on chronic warfarin therapy, and concurrent thrombocytopenia developed progressive anemia during hospitalization. Warfarin was held for a planned procedure, and bridging anticoagulation with therapeutic-dose enoxaparin was initiated. Imaging subsequently identified a 3.5 × 2.4 × 5.5 cm left iliacus muscle hematoma without evidence of trauma or active extravasation. The patient was managed conservatively with serial monitoring and individualized anticoagulation adjustments, resulting in hemoglobin stabilization and clinical improvement.

This case reinforces the multifactorial nature of bleeding risk in patients with malignancy receiving anticoagulation and highlights that INR alone does not fully capture hemorrhagic risk when additional anticoagulants, thrombocytopenia, renal impairment, and cancer-related hemostatic alterations are present.

## Introduction

Patients with active malignancy represent a particularly high-risk population for both thromboembolic events and bleeding complications, with major bleeding rates two to threefold higher than those in non-cancer patients receiving anticoagulation [1,2]. Despite the increasing use of direct oral anticoagulants, warfarin remains an important and appropriate therapeutic option for select patient populations, including those with cost limitations or specific indications such as mechanical heart valves or antiphospholipid syndrome [3,4]. In patients with cancer, anticoagulation management is further complicated by fluctuating drug-drug interactions, poor oral intake, acute illness, treatment-related cytopenias, and impaired hepatic function, all of which contribute to an unpredictable anticoagulant effect that may not be adequately captured by International Normalized Ratio (INR) monitoring alone.

Non-traumatic iliacus and iliopsoas muscle hematomas are uncommon but clinically significant complications of anticoagulation therapy. In a multicenter cohort of 375 patients with major muscular hematomas, 45 (12%) involved the iliopsoas compartment; spontaneous iliopsoas hematomas have been estimated to occur at a rate of 3.8 per 1,000 ICU admissions among patients hospitalized for more than three days; and a systematic review identified 174 reported cases of iliopsoas hematoma with femoral nerve palsy, of which 54% were attributable to pharmacologic anticoagulation [5,6,7]. These hematomas frequently present insidiously, with groin, hip, or flank pain and progressive anemia, and may be complicated by femoral nerve compression in more severe cases. Importantly, observational data suggest that the majority of these events occur despite INR values within or below the therapeutic range, underscoring that INR alone is an insufficient marker of hemorrhagic risk, particularly when additional anticoagulants are concurrently active or when risk factors such as older age, renal impairment, concurrent antiplatelet therapy, and coagulopathy are present [5,6].

Bridging anticoagulation with therapeutic-dose low-molecular-weight heparin represents a particularly high-risk period in cancer patients because its anticoagulant effect is not reflected by INR monitoring and may be amplified by concurrent thrombocytopenia, organ dysfunction, and malignancy-related hemostatic abnormalities.

We present a case of a non-traumatic iliacus muscle hematoma occurring during therapeutic-dose enoxaparin bridging in a patient with active metastatic malignancy, illustrating the multifactorial nature of hemorrhagic risk in this population and the importance of individualized anticoagulation management beyond INR-based assessment.

## Case presentation

A 67-year-old man with a complex medical history, including metastatic squamous cell carcinoma of the lung status post port placement, squamous cell carcinoma of the larynx with cervical lymph node metastasis, congestive heart failure, and atrial flutter on chronic warfarin therapy, was admitted following referral from his outpatient cancer center. He was undergoing active cisplatin-based chemoradiation therapy when routine laboratory evaluation demonstrated elevated bilirubin, and he was noted to be mildly febrile, tachycardic, tachypneic, and short of breath, with progressive dysphagia.

On admission, the patient reported worsening odynophagia and dysphagia in the setting of mucositis, along with generalized weakness. He denied any history of trauma or falls, and no falls or traumatic events were documented during hospitalization. On physical examination, the patient appeared chronically ill but was alert and oriented. Examination of the head and neck revealed mucositis with erythema of the oropharynx without active bleeding. Cardiovascular examination demonstrated a regularly irregular rhythm consistent with his known history of atrial flutter, although an electrocardiogram obtained during hospitalization demonstrated sinus tachycardia with left axis deviation. Pulmonary examination revealed clear breath sounds bilaterally without acute respiratory distress. Abdominal examination was soft and non-tender. Musculoskeletal examination showed no focal tenderness or signs of trauma, and neurologic examination was non-focal. Gastrointestinal and genitourinary bleeding sources were excluded clinically, as the patient denied melena, hematochezia, hematemesis, and hematuria throughout hospitalization, and there was no evidence of overt external bleeding.

Admission laboratory evaluation was notable for hemoglobin 9.1 g/dL, platelet count 114 × 10⁹/L, creatinine 1.77 mg/dL (baseline 1.1 mg/dL, consistent with acute kidney injury of prerenal etiology), aspartate aminotransferase 61 U/L, alanine aminotransferase 53 U/L, alkaline phosphatase 193 U/L, and total bilirubin 2.60 mg/dL, with liver function abnormalities attributed to prior chemotherapy. A sepsis workup was initiated given the patient’s fever and tachycardia; lactate was within normal limits, and blood cultures demonstrated no growth, with no laboratory or clinical evidence of disseminated intravascular coagulation. Anticoagulation was managed through an outpatient anticoagulation clinic. The day prior to admission, his INR was subtherapeutic at 1.3, and he was instructed to take an increased dose of warfarin. He initially declined bridging anticoagulation. During hospitalization, warfarin management was complicated by poor oral intake, acute illness, active malignancy, and a significant drug-drug interaction with amiodarone. Platelet counts demonstrated a downward trend throughout hospitalization, declining from 114 × 10⁹/L on admission to a nadir of approximately 81 × 10⁹/L, consistent with treatment-related myelosuppression. Warfarin was subsequently held on hospital day three in anticipation of a planned procedural intervention. Warfarin was restarted on hospital day five, although the INR remained persistently subtherapeutic, necessitating continued enoxaparin bridging. Bridging anticoagulation with therapeutic-dose enoxaparin (1 mg/kg twice daily, 80 mg every 12 hours) was initiated on hospital day six and continued through hospital day 20, for a total duration of 14 days. Anti-Xa levels were not monitored during the bridging period. Renal function had improved by the time enoxaparin was initiated, with creatinine trending toward baseline, although mild impairment persisted.

Beginning several days after enoxaparin initiation, the patient developed a progressive decline in hemoglobin without an identifiable external source of bleeding. On hospital day 15, the patient reported new-onset left thigh and hip pain, with difficulty bearing weight and pain on movement at the knee and hip, without associated swelling, ecchymosis, or fixed hip flexion deformity. Vital signs at the time of symptom onset were notable for a heart rate of 90 bpm and blood pressure of 123/73 mmHg; the patient remained hemodynamically stable without orthostatic changes. Femoral nerve examination was intact, with no sensory deficits or weakness in the distribution of the femoral nerve documented. The nadir hemoglobin of 6.9 g/dL occurred on hospital day 16, preceding imaging later that day, which identified the hematoma (Figure 1).

**Figure 1:**
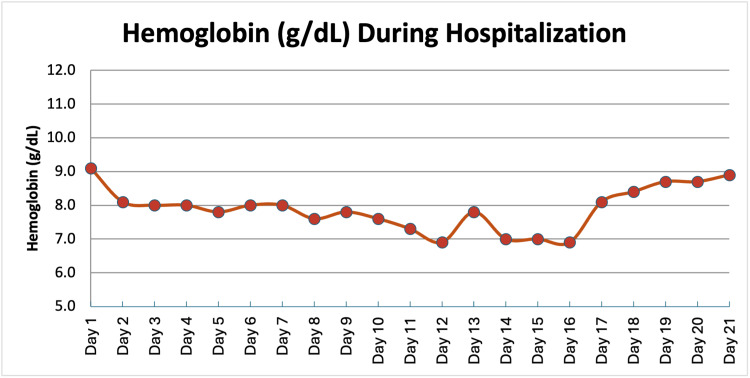
Hemoglobin trend during hospitalization. Hemoglobin demonstrated a progressive decline from 9.1 g/dL on admission to a nadir of 6.9 g/dL on hospital day 16, prompting red blood cell transfusion, with a subsequent increase to 8.2 g/dL and stabilization at 8.1 g/dL.

Contrast-enhanced CT of the pelvis, acquired in the portal venous phase, demonstrated a 3.5 × 2.4 × 5.5 cm hyperdense collection within the left iliacus muscle measuring approximately 62 Hounsfield units, consistent with an acute hematoma (Figure 2). No active arterial contrast extravasation was identified. The hematoma was located medial to the iliac bone and lateral to the psoas muscle, with displacement of the adjacent iliacus muscle fibers but without extension into the retroperitoneal space or compression of the femoral nerve at the level of the inguinal ligament. Imaging of the surrounding osseous structures demonstrated ill-defined sclerosis of the posterior left iliac bone, consistent with known metastatic disease and grossly unchanged from prior imaging, without evidence of tumor-related hemorrhage or pathologic fracture at the hematoma site. At the time of hematoma identification on hospital day 16, the platelet count was 106 × 10⁹/L and creatinine was 0.87 mg/dL, improved from the admission value of 1.77 mg/dL and below the documented baseline of 1.1 mg/dL.

**Figure 2:**
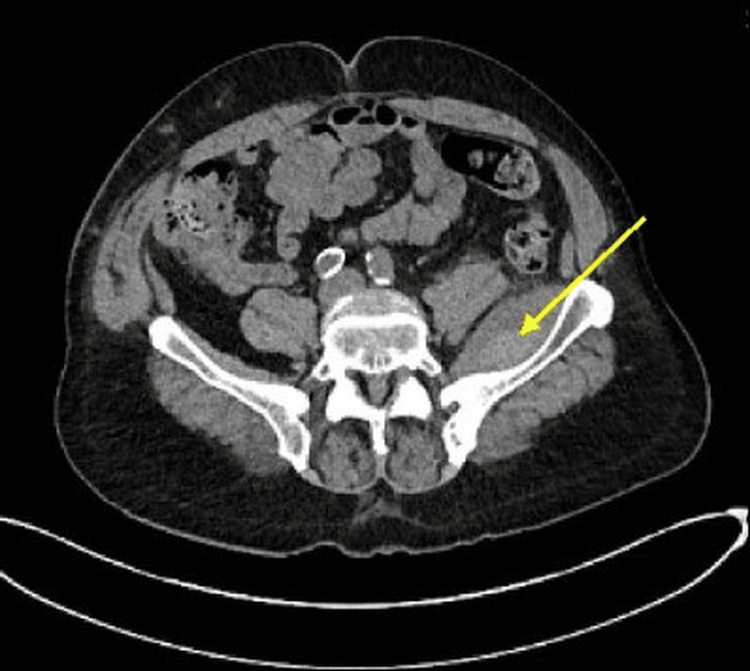
Contrast-enhanced computed tomography of the pelvis (portal venous phase, axial view). A 3.5 × 2.4 × 5.5 cm hyperdense collection, measuring approximately 62 Hounsfield units, is identified within the left iliacus muscle (arrow), consistent with an acute hematoma. The hematoma is located medial to the iliac bone and lateral to the psoas muscle, without significant extension beyond the iliacus compartment or active contrast extravasation.

Laboratory evaluation demonstrated a progressive decline in hemoglobin during hospitalization without evidence of overt bleeding (Table 1, Figure 1, and Figure 3). Hemoglobin decreased from 9.1 g/dL on admission to 6.9 g/dL on hospital day 16. INR values during the period of hemoglobin decline ranged from 1.18 to 1.43, remaining subtherapeutic, although it is important to note that INR does not reflect the anticoagulant activity of low-molecular-weight heparin. The patient subsequently received a packed red blood cell transfusion, with hemoglobin increasing to 8.2 g/dL later that day and remaining stable at 8.1 g/dL the following morning.

**Table 1:** Hemoglobin, INR, platelet count, creatinine, and major clinical events during hospitalization. Hemoglobin progressively declined from 9.1 g/dL on admission to a nadir of 6.9 g/dL on hospital day 16, prompting RBC transfusion with subsequent stabilization. Warfarin was temporarily held for a planned procedural intervention, and therapeutic-dose enoxaparin bridging was later initiated when the INR remained persistently subtherapeutic after warfarin was restarted. Concurrent thrombocytopenia, fluctuating renal function, and new-onset hip/thigh pain preceded computed tomography identification of the iliacus hematoma. INR: International normalized ratio.

Hospital Day	Hemoglobin (g/dL)	INR	Platelet Count (×10⁹/L)	Creatinine (mg/dL)	Clinical Context
Day 1 (Admission)	9.1	1.3	114	1.77	Admission; subtherapeutic INR
Day 2	8.6	1.35-1.40	100	1.53	INR rising; warfarin dose adjusted
Day 3	8.3	2.73	104	1.16	Therapeutic INR; warfarin held for planned procedure
Day 4	8.1	2.37	96	1.25	INR trending downward
Day 5	8.0	1.65	93	1.15	Warfarin restarted; INR subtherapeutic
Day 6	7.8	-	111	1.03	Enoxaparin 80 mg every 12 hours started
Day 13	7.8	1.18	86	1.27	Subtherapeutic INR; enoxaparin active
Day 15	7.0	1.23	99	0.92	New left thigh/hip pain noted
Day 16	6.9	1.33	106	0.87	Nadir hemoglobin; RBC transfusion; CT showed hematoma
Day 16 (post-transfusion)	8.2	-	112	0.9	Post-transfusion hemoglobin
Day 17	8.1	1.43	109	0.92	Hemoglobin stabilization

**Figure 3:**
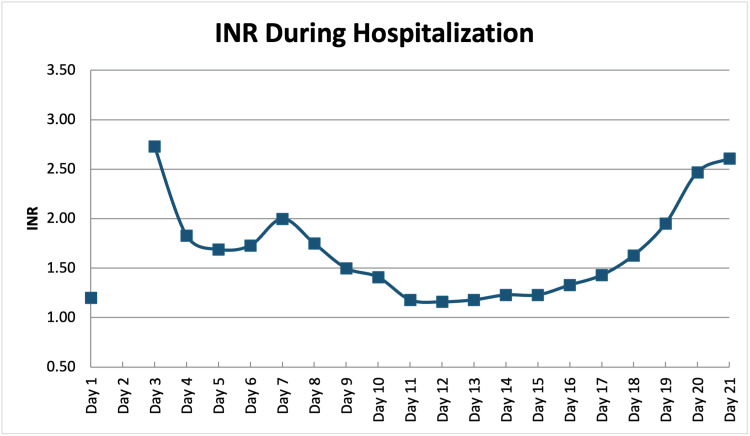
INR trend during hospitalization. INR values fluctuated during hospitalization, ranging from subtherapeutic to therapeutic levels. During the period of hemoglobin decline and at the time of hematoma detection, INR values remained subtherapeutic, ranging from 1.18 to 1.43. INR: International normalized ratio.

Given the patient’s elevated thromboembolic risk from atrial flutter, hemodynamic stability, and absence of active extravasation on imaging, anticoagulation was continued with careful dose adjustment and close monitoring following discussion with the multidisciplinary team. Interventional radiology was consulted and recommended against drainage given the absence of a drainable fluid component and elevated procedural bleeding risk in the setting of thrombocytopenia and ongoing anticoagulation. The patient was managed conservatively with serial hemoglobin monitoring, clinical observation, and individualized anticoagulation adjustment under pharmacy guidance. No anticoagulation reversal agents were administered, and neither surgical intervention nor interventional radiology procedures were performed.

The patient remained hemodynamically stable throughout hospitalization. Hemoglobin levels stabilized with conservative management, and no progression of the hematoma was observed clinically. Anticoagulation management was individualized based on thromboembolic risk, bleeding severity, renal function, and overall oncologic prognosis, with warfarin subsequently titrated to achieve and maintain a therapeutic INR prior to discharge.

## Discussion

Non-traumatic iliacus and iliopsoas hematomas are uncommon but potentially serious complications of anticoagulation therapy that often present insidiously with progressive anemia and groin, hip, or flank pain [5,6,7]. Observational studies have demonstrated these hematomas across a range of anticoagulation intensities, suggesting that hemorrhage is frequently driven by multiple concurrent risk factors rather than anticoagulation intensity alone.

In this case, the temporal relationship between therapeutic-dose enoxaparin initiation and subsequent hemoglobin decline most strongly implicates bridging anticoagulation as the primary pharmacologic contributor to hemorrhage. Importantly, INR does not reflect low-molecular-weight heparin activity, and the subtherapeutic INR values observed during this period reflected warfarin effect only. Anti-Xa levels, which more appropriately assess enoxaparin activity, were not obtained and represent a limitation of this report.

Earlier in the admission, prior to enoxaparin initiation, the INR reached 2.73, within the therapeutic range for atrial flutter, while hemoglobin had already begun declining. This early hemoglobin decrease, occurring in the absence of supratherapeutic anticoagulation, likely reflected the combined effects of malignancy, systemic illness, and treatment-related myelosuppression, further illustrating the difficulty of attributing anemia to a single etiology in this population and underscoring that bleeding risk extends beyond anticoagulation intensity.

Bleeding risk was further amplified by progressive thrombocytopenia, renal impairment, hepatic dysfunction, active malignancy, and concurrent amiodarone therapy. Platelet counts declined throughout hospitalization, consistent with treatment-related myelosuppression, impairing primary hemostasis independently of anticoagulation intensity. Renal dysfunction may also have prolonged enoxaparin activity through impaired clearance, while elevated bilirubin and transaminases suggested hepatic dysfunction that may have compounded coagulopathy. Drug-drug interactions further complicated anticoagulation management in this patient. Amiodarone inhibits CYP2C9 and CYP3A4, the primary enzymes responsible for warfarin metabolism, and may increase warfarin effect by approximately 30-50% within the first several weeks of initiation [8]. In the setting of acute illness, poor oral intake, and variable vitamin K exposure, this interaction may contribute to instability between measured INR and overall anticoagulant effect.

Recent radiologic studies suggest that active contrast extravasation is the strongest imaging predictor of mortality in iliopsoas hemorrhage [9]. In this patient, the absence of active extravasation supported conservative management. In the absence of active extravasation or hemodynamic compromise, conservative management is supported by observational studies demonstrating successful non-operative treatment in many iliopsoas hematomas [7]. Contemporary cancer-associated thrombosis guidelines recommend individualized assessment of thromboembolic risk, bleeding severity, imaging findings, and overall oncologic prognosis when making anticoagulation decisions in patients [10]. Given the patient’s hemodynamic stability and elevated thromboembolic risk, anticoagulation was continued with individualized dose adjustment and close monitoring rather than reversal or invasive intervention.

This case highlights the multifactorial nature of hemorrhagic risk in anticoagulated cancer patients and emphasizes the importance of maintaining suspicion for occult muscular hemorrhage in patients who develop unexplained anemia or new musculoskeletal symptoms. It also demonstrates that standard coagulation monitoring, including INR, does not fully reflect bleeding risk when multiple anticoagulants and concurrent hemostatic abnormalities are present.

## Conclusions

This case demonstrates that non-traumatic muscular hematoma in anticoagulated patients with active malignancy is a multifactorial complication driven by the convergence of therapeutic bridging anticoagulation, progressive thrombocytopenia, renal impairment, hepatic dysfunction, drug-drug interactions, and cancer-related hemostatic alterations. The cumulative hemorrhagic risk imposed by these concurrent factors may substantially exceed what any single laboratory parameter suggests, and standard coagulation monitoring, including INR, may not capture the full anticoagulant burden when multiple agents or hemostatic insults are present concurrently. Clinicians should maintain a high index of suspicion for occult muscular hemorrhage in anticoagulated cancer patients who develop unexplained anemia or new musculoskeletal symptoms and should pursue early cross-sectional imaging when clinical suspicion arises. Individualized anticoagulation management, incorporating assessment of renal function, platelet trends, drug interactions, and oncologic status, is essential to optimize outcomes in this complex population.
